# Clinical Report on Method of Operating

**Published:** 1897-10

**Authors:** H. C. Register

**Affiliations:** Philadelphia


### CLINICAL REPORT ON METHOD OF OPERATING.[1]

BY DR. H. C. REGISTER, PHILADELPHIA.

INDIVIDUAL methods of operating are of minor importance if they do not possess a qualifying reason why they are so done, embodying a "principle" that applies in all like conditions. That

[1] Read before the Academy of Stomatology, March 23, 1897.

which is involved, as a principle, in the manipulation of gold as performed by myself on Saturday last, consists in adapting gold in regulated lamina, in opposition to an irregular form of cohesive work, and the fixation by forcing it to place by strips of bibulous paper or old soft linen, thus making a tight joint by packing the gold to every point of contact by the same effort, in opposition to retainers, cohesive and otherwise, which when done by instrument points are more likely to let some inequality remain, and also wound the finished tooth tissue, which is detrimental to the saving quality of the filling so far as it makes an inequality, thus preventing the hardening of the dentine beneath it.

The calcification of the fibrillæ, I claim, always takes place in forming a zone of secondary tissue in a filled tooth when the mechanical and physical relations are made compatible with each other to the exclusion of recurring exciting causes of caries. Metal fillings, through their thermal irritation, produce that result sooner than the zinc or gutta-percha. To this end the formation of cavities should conform to the natural cleavage of the enamel, which radiates from the stratum granulosum to the periphery, and, after Williams, are held together by the interprismatic cementing substance. An enamel wall, under the best conditions for its future preservation, should stand in this relation to the filling, the length of the enamel prisms, where possible, reaching from the enamel cuticle to the stratum granulosum, with the surface made smooth. In all cavities thus formed we get, as an anatomical result, a V-shape to a greater or less extent, this being always natural as applied to enamel.

In connection with this, and all other forms of cavities, I cannot lay too much importance upon the sterilizing and alkaline influence of bathing the cavities with iodine, and decomposing it and all carbohydrates and starchy matter, including germ-life, which is in the main composed of the latter material, and of placing the inner enamel and dentine in an alkaline or neutral condition before filling. The changes in the tissue, I believe, are as great as they are apparent, permitting and producing an antithermal change through secondary dentine more quickly, and anticipating the action of germ-life that may be left in the tissues.

The use of a matrix in connection with this method, and all others as well, should stand as a necessity only, in giving expression to the cavity,—*never a positive, fixed, artificial wall!*

As the environment of the tooth will be the exciting cause of future caries, the filling must anticipate, so far as possible, the dissolution of the interprismatic cement or the calcoglobulin, as pointed out by Williams, before a recurrence of decay can take place in any part of the filled border properly protected by the gold. To do this best is the aim of all honest operators. I present this method not as one of an hour, a day, or a month, but one that has stood the clinical criticism of fourteen years as a system, with the good work continuing to preserve badly broken-down teeth, as observed from carefully kept charts that tell the conditions then and now.

When one or more of the walls are broken, a matrix becomes a necessity, and here planished copper or German silver works best with me,—always fitting it to the individual case. This may be done by soft soldering the ends or using the silk ligature, which I generally prefer, winding it round and round the matrix to its width, which comes to the bulbous portion of the tooth only. To planished copper, which is rendered so pliable and dead by annealing, I give the preference for making all kinds of matrices.

If it is desired to give the filling an oval form or contour at the knuckling point of contact, it is only necessary to hold the copper strip, before cutting, in the palm of the hand or some fairly soft surface and burnish it to the extent required. The ligature takes it to place uniformly throughout the boundary line and leaves a mould to be replaced by the filling.

Gold cylinders, the length of which correspond to the cross-axis of the cavity, are now laid on the bottom and sides and pressed to place by bibulous paper folded once, or as many times as will correspond with the work in hand; and soft linen cut into strips is to be used in like manner without folding, and in its preparation must always be cut, never torn. After the gold has been pressed to place under paper by "plugging pliers" (a special form is best), the ends of the cylinders are *turned over* and *around the cavity*, *between* the *tooth* and the *matrix*, or the first cylinders may be placed and adapted under the bibulous paper, the edges of these turned over the border of the cavity at the cervix, and the matrix then applied as described, pressing against the cylinders, the operation being continued by the use of mat gold and malleted to place with the paper upon the gold, followed after its removal with proper small, smooth points until all parts of the boundary, as you progress, are filled *without the instrument coming in contact with tooth-structure.* After the enamel has been thus protected, to a greater or less degree, not being so essential as with dentine, the cohesive gold can be added wherever the masticating surface or contour calls for it. In the larger restorations the borders of the filling should be composed of cylinders carefully laid in contact with dentine and enamel throughout the joint contact in lamina. The centre or body may be filled with mat or crystal gold, as the air-spaces in the work are in no way detrimental. The time thus gained is all secured by the use of soft gold, it permitting of about two-thirds of the cavity being filled in one-third the time, as a gain over cohesive work.

No special retaining device, as a rule, is necessary, other than a slight irregularity that may naturally exist; otherwise undercuts should be slight, and to see that the pounding and triturating forces of mastication are met by the cylinders being anchored as much within the body of the tooth as extending beyond the cavity in restoring the contour to any required form.

This method of operating, I believe, is in direct line with the latest discovery in histological research in its relation with the several anatomical parts of tooth-structure, both mechanically and physically related, and as a protection from the microgerm environment. Thus do we find non-cohesive or soft gold laid in lamina in juxtaposition against dentine and enamel, in contradistinction to small pieces being added cohesively in retainers, to be no veneer but of better grain, and being opposite in all its relations as a tooth-preserver, both physically and mechanically.

